# Preference for Averaging in East Asian Faces: A Source of Potential Guidance in Aesthetic Plastic Surgery

**DOI:** 10.1093/asjof/ojad058

**Published:** 2023-07-11

**Authors:** Cyrus Steppe, Richard Cinclair, Edward Yuan Wen, Al Aly

## Abstract

**Background:**

Relatively little research has been done on the application of objective tools in guiding Ethnic Plastic Surgery in Asian patients. The evolutionary psychology theory of koinophilia, or love of average features, presents the basis for a solution to build a foundation for crowd-sourced East Asian aesthetic standards.

**Objectives:**

The authors hypothesize that the averaged composite face in a cohort will be viewed as significantly more attractive than their respective cohort.

**Methods:**

Cohorts were created based on the gender of the individual in the photograph (40 females and 40 males of East Asian descent). Two surveys were created, 1 for the female cohort and the other for the male. The surveys assessed the aesthetic preference of each photograph using a Likert scale ranging from 1 to 7. Surveys were distributed using the popular crowdsourcing program Amazon Mechanical Turk (Amazon, Seattle, WA).

**Results:**

The authors received 875 respondents for the male cohort survey and 876 respondents for the female cohort survey. For both the female and male cohorts, the composite images had a statistically significantly higher rating (*P* < .001) than the mean of the other images. Among other significant demographic findings, when considering both ethnicity and location of residence, Asian raters living in Asia preferred the composite significantly more than Asian raters living in North America (*P* < .001).

**Conclusions:**

Raters’ preference for the composite average face is in concordance with the evolutionary psychology literature. Thus, this study affirms the utility of using facial composites to guide surgeons in identifying aesthetic standards for patients of East Asian descent.

Aesthetic surgeries, such as blepharoplasties, rhinoplasties, and mandibular augmentations, have become increasingly popular in East Asian countries.^1-3^ Despite evident regional differences, relatively little research has been done on the application of objective tools to guide Ethnic Plastic Surgery in Asian patients. Surgeons have begun to highlight the importance of abandoning certain cannons of aesthetics that have been catered to treating Caucasians when consulting patients of East Asian descent. The application of Caucasian-based ideals of beauty may contribute to a loss of cultural identity and subsequent Westernization of appearance.^2,4,5^ A patient's expectations should always be one of the most important components when setting preoperative goals. A surgeon equipped with objective criteria, or *yardsticks*, of attraction as well as an understanding of cultural preference has an advantage in guiding and delivering results that increase patient satisfaction.^6^

The field of evolutionary psychology has demonstrated that the theory of koinophilia is a reasonable approach to determining attractiveness in all species including humans. Koinophilia, or the preference for mates with the most average features, is a thoroughly studied concept, producing a plethora of research on attraction.^7-9^ The closer a person's anatomy is to the average, or composite, of a large cohort, the more attractive that individual tends to be.^7^ This theory was recently confirmed in a study that we conducted on Caucasian male and female cohorts where the composite of 40 individuals of each gender was found to be more attractive than any individual who contributed to the composite.^6^

Theoretically, the same should hold for other ethnic groups.^9^ However, a separate psychological phenomenon in beauty, “cognitive averaging,” may play a role when evaluating a minority ethnicity.^10^ Cognitive averaging allows humans to average over time. In other words, we continuously average who we see and thus are affected by what we recently have been exposed to, and less so by what we have seen in the past. A practical example comes from the fashion industry; a new clothing style may seem unusual or unappealing at first but after repeated exposure, often with the help of celebrities, we become accustomed and eventually fond of the trend. Langlois et al connects this concept to our attraction of particular faces. We favor faces that are more familiar and similar to what we have been exposed to.^8^ The demographics of a person's community may influence preference dramatically, as varying exposure could create a cognitive average that is different from an ethnicity's anatomical average. We will question the effect of cognitive averaging by comparing regions where people of East Asian decent are the majority to those regions where they are the minority.

This study is part of a series of experiments on different ethnic groups that will include African Americans, Latinos, and East Asians. The purpose of this study is to test tenants of koinophilia and introduce “cognitive averaging” in an East Asian population and determine if the theories translate to this ethnic group.

## METHODS

### Data Collection

The Chicago Face Database permitted the use of 80 (40 females and 40 males) standardized, front-facing photographs of people of East Asian descent. There are 57 female and 52 male photographs of East Asian decent in the database, 40 of each gender were randomly selected for the study. The Chicago face database was chosen because of the quality, standardization, size, and diversity of their image sets. Cohorts were created based on the gender of the individual in the photograph. Faces were mapped, measured, and averaged using the web-based program Webmorph.org (Glasgow, Scotland). One hundred and eighty-eight individual points were manually placed on every face, with each corresponding to the location of a specific feature (eg, center of the iris). Webmorph.org was additionally used to create a composite image for each sex, consisting of the 40 real images within that cohort.

Two surveys were created, 1 for the female cohort and the other for the male, using Google Forms. The surveys consisted of 41 seven-point Likert scale questions, ranging from a rating of 1 being the least attractive to 7 being the most attractive. Each question presented the respondent with a different face from the cohort. Surveys were advertised and distributed using the popular crowdsourcing program Amazon Mechanical Turk (MTurk; Amazon, Seattle, WA). After successful completion of the survey, evaluated both by the submission of the correct completion code administered at the end of the survey and by time-to-completion, respondents were compensated for their time. Demographic information, including age, race, country of residence, gender, and sexual preference, were all collected in addition to the image ratings.

### Statistical Analysis

**Figure 1.**
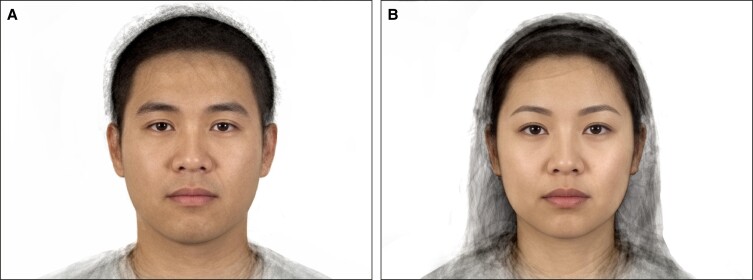
The (A) male composite images (created from averaging the 40 real images in the male cohort) and (B) female composite images (created from averaging the 40 real images in female the cohort).

The 40 real images were compared to the composite image for the male and female (Figure 1) cohorts by utilizing *t* tests. The difference between the mean rating of the composite and real images (Composite-Real Difference) was used to assess the strength of the preference for the composite over the real images. The Composite-Real Difference was compared across rater demographics (ie, age, gender, ethnicity, and country of residence) with *t* tests. Shapiro–Wilk normality tests were performed for 40 averaged scores (*P* > .05 presents qualified normal distribution). Demographic subgroupings were additionally evaluated with *t* tests.

## RESULTS

### Ratings

We received 875 respondents for the male cohort survey and 876 respondents for the female cohort survey. The average age of the male cohort was 30.31 years old (range, 21-56 years of age), and the average age of the female cohort was 27.65 years old (range, 19-46 years of age). Shapiro–Wilk normality tests of the 40 real models in each cohort revealed a normal distribution (*P* > .05).

For the male cohort, the composite images (mean = 4.61) had a statistically significantly higher rating (*P* < .0001) than the other images (mean = 3.63). Furthermore, voters from each age range, gender, and country of residence rated the male composite image significantly higher than the other male images (all *P* < .0001; Table 1). One male face was scored as significantly more attractive (*P* < .0001) than the rest of the cohort, and more attractive than the composite (mean = 4.97). Next, we compared the preference of the composite (ie, the difference between the mean rating of the composite and the mean rating of the real individuals) for each demographic (Table 2). We found that 18- to 29-year-old respondents preferred the composite significantly <30- to 39-, 40- to 49-, and 60+-year-old respondents (all *P* < .05). Furthermore, we found that 60+-year-old respondents also preferred the composite significantly >40- to 49- and 50- to 59-year-old respondents. Across all other age groups, we found that raters found the composite similarly more attractive than the real images (all *P* < .05). Female voters preferred the male composite image the most strongly, significantly more than male (*P* = .0383) and nonbinary (ie, those who do not identify as male or female) voters (*P* < .0001). Furthermore, nonbinary voters preferred the composite the least strongly, significantly less than both males and females (both *P* < .0001). Country of residence was found to significantly correlate with Composite-Real Difference, with only Asians and North Americans having similar differences (*P* = .2779). Raters from Europe preferred the composite the most, significantly more than raters from North America, Asia, and South America (all *P* < .05). We found that North American voters preferred the composite the least relative to voters from other countries, significantly less than South Americans, Asians, and Europeans (all *P* < .05). When considering both ethnicity and location of residence, Asians living in Asia preferred the composite significantly more than Asians living in North America (*P* < .001).

**Table 1.** Real Images vs Composite Perceived Attractiveness of Male Cohort

Respondent demographics	Respondents (*n*)	Rating of real images (mean [SD])	Rating of composite (mean [SD])	*P*-value
All	875	3.63 [1.67]	4.61 [1.52]	<.001
Age (years) 18-29 30-39 40-49 50-59 60+	275 326 165 73 36	3.58 [1.70] 3.61 [1.68] 3.72 [1.72] 3.7 [1.40] 3.65 [1.51]	4.34 [1.69] 4.74 [1.43] 4.74 [1.43] 4.64 [1.31] 4.92 [1.36]	<.001 <.001 <.001 <.001 <.001
Gender Male Female Nonbinary	432 414 29	3.74 [1.67] 3.47 [1.67] 4.18 [1.43]	4.63 [1.54] 4.61 [1.49] 4.52 [1.55]	<.001 <.001 <.001
Country of residence North America South America Asia Europe	489 73 156 127	3.5 [1.61] 3.25 [1.78] 3.77 [1.65] 4.05 [1.73]	4.47 [1.50] 4.64 [1.55] 4.88 [1.51] 4.78 [1.51]	<.001 <.001 <.001 <.001
Ethnicity Asian Black or African American Caucasian Hispanic, Latino, or of Spanish Origin Other	220 50 470 63 72	3.54 [1.59] 3.40 [1.72] 3.64 [1.99] 3.93 [1.99] 3.74 [1.63]	4.64 [1.47] 4.72 [1.58] 4.62 [1.49] 4.62 [1.62] 4.47 [1.67]	<.001 <.001 <.001 <.001 <.001

SD, standard deviation.

**Table 2.** Male Cohort: Comparison of Perceived Attractiveness of Composite Compared to Real Images Across Respondent Demographics

Respondent demographics	Respondents (*n*)	Mean rating of all images	Mean rating of composite	30-39 years (*P*-value)	40-49 years (*P*-value)	50-59 years (*P*-value)	60+ years (*P*-value)	Female (*P*-value)	Nonbinary (*P*-value)	Asia (*P*-value)	Europe (*P*-value)	South America (*P*-value)	Hispanic, Latino, or of Spanish origin (*P*-value)	Native American (*P*-value)	Asian (*P*-value)	Black or African American (*P*-value)	Other (*P*-value)
All raters	875	3.63	4.61	—	—	—	—	—	—	—	—	—	—	—	—	—	—
Age (years) 18-29 30-39 40-49 50-59 60+	275 326 165 73 36	3.58 3.61 3.72 3.7 3.65	4.34 4.74 4.74 4.64 4.92	.0025 — — — —	.0208 .3651 — — —	.119 .1151 .4613 — —	.0001 .2989 .0475 .0091 —	— — — — —	— — — — —	— — — — —	— — — — —	— — — — —	— — — — —	— — — — —	— — — — —	— — — — —	— — — — —
Gender Male Female Nonbinary	432 414 29	3.74 3.47 4.18	4.63 4.61 4.52	— — —	— — —	— — —	— — —	.0383 — —	<.0001 <.0001 —	— — —	— — —	— — —	— — —	— — —	— — —	— — —	— — —
Country of residence North America Asia Europe South America	127 489 73 156	4.05 3.5 3.25 3.77	4.78 4.47 4.64 4.88	— — — —	— — — —	— — — —	— — — —	— — — —	— — — —	.0313 — — —	<.0001 .0015 — —	.0014 .2779 .035 —	— — — —	— — — —	— — — —	— — — —	— — — —
Ethnicity Caucasian Hispanic, Latino, or of Spanish Origin Native American Asian Black or African American Other	469 63 34 219 50 40	3.64 3.93 3.97 3.53 3.4 3.53	4.62 4.63 4.62 4.63 4.72 4.3	— — — — — —	— — — — — —	— — — — — —	— — — — — —	— — — — — —	— — — — — —	— — — — — —	— — — — — —	— — — — — —	.0133 — —— — — —	.0108 .6516 — — — —	.3327 .0007 .0008 — — —	.0065 <.0001 <.0001 .0819 — —	.0911 .5497 .3559 .0101 <.0001 —

For the female cohort, the composite images (mean = 5.11) had a statistically significantly higher rating (*P* < .0001) than the other images (mean = 3.76). The respondents were broken up by age range, gender, and home region. Raters of each age range, gender, and country of residence rated the female composite image significantly higher than the other female images (all *P* < .0001; Table 3). Next, we compared the preference of the composite over the real image across the demographics (Table 4). Eighteen to 29-year-old respondents preferred the composite the least strongly, significantly <40 to 49 and 60+-year-old respondents (both *P* < .05). Respondents >60 years old preferred the composite the most strongly, significantly >50 to 59, 30 to 39, and 18 to 29-year-old voters (all *P* < .05). Once again, nonbinary raters preferred the composite the least strongly again, significantly less than male and female voters (both *P* < .05). Male voters preferred the composite the most, significantly more than nonbinary and female respondents (both *P* < .05). Once more, country of residence was found to significantly correlate with Composite-Real Differences (all *P* < .05) except with only North and South Americans having similar Composite-Real Differences (*P* = .2355). This time, we found that Asian respondents preferred the composite the most, significantly more than North American, European, and South American voters (all *P* < .05). Lastly, Europeans preferred the female composite the least, significantly less than North Americans, South Americans, and Asians (all *P* < .05).

**Table 3.** Real Images vs Composite Perceived Attractiveness of Female Cohort

Respondent demographics	Respondents (n)	Rating of real images (mean [SD])	Rating of composite (mean [SD])	*P*-Value
All	876	3.76 [1.65]	5.11 [1.46]	<.001
Age (years) 18-29 30-39 40-49 50-59 60+	273 325 161 80 31	3.7 [1.67] 3.79 [1.67] 3.69 [1.69] 4.02 [1.44] 3.73 [1.57]	4.92 [1.50] 5.12 [1.52] 5.19 [1.48] 5.34 [1.16] 5.32 [1.09]	<.001 <.001 <.001 <.001 <.001
Gender Male Female Nonbinary	420 440 16	3.6 [1.65] 3.89 [1.65] 4.47 [1.59]	5.07 [1.44] 5.12 [1.47] 5.56 [1.26]	<.001 <.001 <.001
Country of residence North America South America Asia Europe	491 84 134 141	3.62 [1.66] 3.41 [1.75] 4.19 [1.65] 3.94 [1.58]	5.05 [1.46] 5.04 [1.47] 5.17 [1.57] 5.23 [1.36]	<.001 <.001 <.001 <.001
Ethnicity Asian Black or African American Caucasian Hispanic, Latino, or of Spanish origin Other	212 56 469 72 67	3.65 [1.52] 3.78 [1.74] 3.77 [1.66] 4.00 [1.88] 3.77 [1.55]	5.18 [1.46] 4.96 [1.57] 5.10 [1.41] 5.24 [1.41] 4.90 [1.61]	<.001 <.001 <.001 <.001 <.001

SD, standard deviation.

**Table 4.** Female Cohort: Comparison of Perceived Attractiveness of Composite Compared to Real Images Across Respondent Demographics

Respondent demographics	Respondents (*n*)	Mean rating of all images	Mean rating of composite	30-39 years (*P*-value)	40-49 years (*P*-value)	50-59 years (*P*-value)	60+ years (*P*-value)	Female (*P*-value)	Nonbinary (*P*-value)	Asia (*P*-value)	Europe (*P*-value)	South America (*P*-value)	Hispanic, Latino, or of Spanish origin (*P*-value)	Native American (*P*-value)	Asian (*P*-value)	Black or African American (*P*-value)	Other (*P*-value)
All raters	876	3.76	5.11	—	—	—	—	—	—	—	—	—	—	—	—	—	—
Age (years) 18-29 30-39 40-49 50-59 60+	273 325 161 80 31	3.7 3.79 3.69 4.02 3.73	4.92 5.12 5.19 5.34 5.32	.3471 — — — —	.0137 .1282 — — —	.3738 .9338 .0976 — —	.0032 .0372 .4862 .0265 —	— — — — —	— — — — —	— — — — —	— — — — —	— — — — —	— — — — —	— — — — —	— — — — —	— — — — —	— — — — —
Gender Male Female Nonbinary	420 440 16	3.6 3.89 4.47	5.07 5.12 5.56	— — —	— — —	— — —	— — —	.0411 — —	.0028 .2194 —	— — —	— — —	— — —	— — —	— — —	— — —	— — —	— — —
Country of residence North America Asia Europe South America	491 84 134 141	3.62 3.41 4.19 3.94	5.05 5.04 5.17 5.23	— — — —	— — — —	— — — —	— — — —	— — — —	— — — —	.1428 — — —	<.0001 <.0001 — —	.2355 .0084 .0017 —	— — — —	— — — —	— — — —	— — — —	— — — —
Ethnicity Caucasian Hispanic, Latino, or of Spanish origin Native American Asian Black or African American Other	468 72 29 211 56 40	3.77 4.01 4.32 3.66 3.79 3.4	5.1 5.24 5.07 5.18 4.96 4.78	— — — — — —	— — — — — —	— — — — — —	— — — — — —	— — — — — —	— — — — — —	— — — — — —	— — — — — —	— — — — — —	.37 — — — — —	<.0001 <.0001 — — — —	.1133 .0107 <.0001 — — —	.1492 .5608 <.0001 .002 — —	.6809 .1807 <.0001 .2332 .0588 —

## DISCUSSION

This study confirms the evolutionary psychology principle of koinophilia in determining its effectiveness in the understanding of beauty and attractiveness in people of East Asian Descent. The findings are similar in nature to those found when the same methodology was utilized in a Caucasian cohort.^6^ It is important to place this study, and others similar to it, in the greater context of the study of beauty and attractiveness. It is likely that koinophilia, or the “love of the average,” is part of a much more complex system that we are starting to learn about. This is akin to the study of the atom in physics, where the discovery of electrons and their characteristics was, and still is, an essential component of our understanding of the atom, but subsequent discoveries have elucidated the existence of many more particles (neutrons, quarks, etc) that add to the overall understanding of the atom and how it functions. Similarly, in the case of beauty and attractiveness, koinophilia and “cognitive averaging”^8,10,11^ are going to combine to give us a better understanding of the subject.

Our research group is in the midst of confirming these findings in other groups, such as African Americans and Latinos, which should further our understanding not only within each group but also for beauty and attractiveness in general. Ultimately, the aim of this study, and others like it, is to give the plastic surgeon an idea of what the “ideal normal” is, as in creating “yard sticks,” both to aim for at surgery and to compare to after surgery. To relate this to a current clinical example of how this works, studies over decades have determined that the nasolabial angle in a Caucasian female nose ideally is between 95° and 100°.^12,13^ Prior to surgery, the surgeon has a yardstick of that angle to aim for at surgery. After surgery, the angle is measured and compared not only to the original nasolabial angle but also to the yardstick, which delineates an objective measure of success/failure.

In this study, crowdsourcing served as the primary mode of evaluating the koinophilic principles in a large sample population. The technique allowed us to survey a large number of East Asian individuals living in both North America and Asia. Of the 1751 respondents to our 2 surveys, 24.6% were Asian, equipping us to study the trends in attraction among people of the same general ethnicity as our cohort.

**Figure 2.**
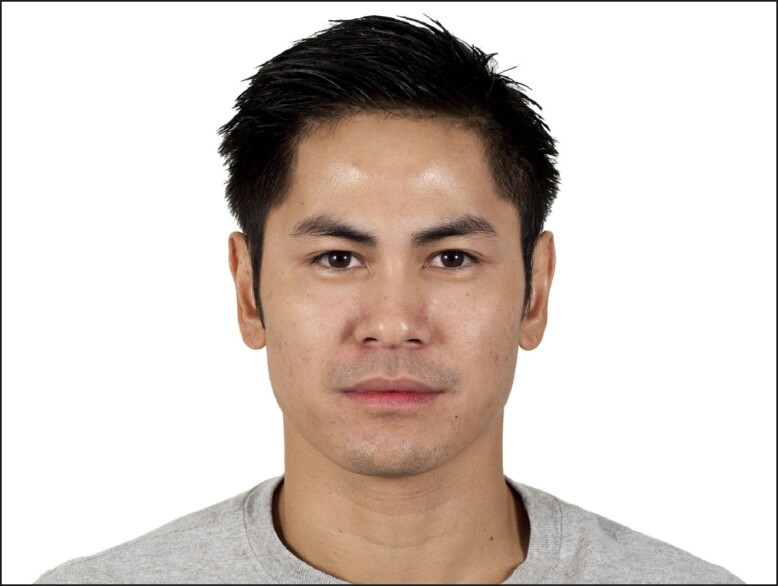
Photograph of the male face that had a significantly higher rating than the composite male image. Published with permission from Ma, Correll, and Wittenbrink (2015). The Chicago Face Database: A Free Stimulus Set of Faces and Norming Data. *Behavior Research Methods 47*, 1122-1135.

Similar to Amaya et al, where a Caucasian cohort was examined, this study found that raters, irrespective of race, gender, sexual orientation, income, or regional setting, rated the composite image as significantly more attractive than the combined mean rating of the rest of the cohort.^6^ However, in this assessment, there was an outlier. In the male cohort, there was 1 face that scored significantly higher than both the composite and the rest of the cohort. Despite reaffirming our hypothesis of a general preference for the average, the favorability of a noncomposite face poses important questions surrounding koinophilia and the complexity of understanding beauty and attractiveness. To that end, attraction to features that are not average has been evaluated in the field of evolutionary psychology.^14-17^ Youthfulness, for instance, seems to be unrelated to averageness and tends to peak in a person's 20s before visible hallmarks of aging emerge.^15^ In this study, the highest rated real females and males were 24 and 28 years old, respectively, both younger than the average of the cohort. Other features that humans favor that are not average, like the sexually dimorphic quality of a strong jawline in males, are notable departures from the principles of koinophilia.^18^ Additionally, the composite in our male cohort may have been too average.^14^ Sharabi et al found that generating a composite from faces that were rated in the top 10% most attractive was more attractive than a composite created using an entire cohort.^11^ Their conclusions potentially explain why our male composite was not the most attractive. Qualitatively, this noncomposite male does have a combination of more masculine and more western features when compared with the composite (Figure 2): a stronger jawline, more pronounced brow, and a sharper nose.^18,19^ The preference for western features in an East Asian face could point to the influence of regional demographics and social media on the construction of a cognitive average. The prevalence of western culture in social media and the global entertainment industry likely acts as a facial fashion runway, to employ a previously used example, in guiding who we find attractive.^20^ Consistent exposure to western celebrities and influencers creates a cognitive average that has more Caucasian features, even if Caucasians are not the majority in a region. This may be the reason why Figure 2 was preferred more than the anatomical composite in Figure 1.

To address this discrepancy in our own findings, we analyzed the regional preference for the composite crossed with the ethnicity of the rater. The logic being that raters of a given ethnicity, in this case Asian, would vary in their preference for a composite depending on the majority ethnicity of that region. Asian respondents in Asia were found to rate the composite significantly higher than Asian respondents in North America. The composite images had more features that aligned with those conventionally preferred by people of East Asian descent.^1,19,21^ For example, both composites in our study possessed a supratarsal crease^3,22,23^ and had a more rounded lower third of the face.^3,21,24^ The consistency of our composites with East Asian beauty standards reinforces the potential of composite images acting as surgical yardsticks.

Regions where Caucasian populations are the majority do not have the same standards of beauty.^5,19,25^ This may explain why North Americans rated the composite as significantly less attractive than the other regions in the study. Additional demographic-centric findings in our study have been explored by other authors. Respondents >60 years old rated the average the highest among other age groups and significantly >18 to 29 year olds. This may be explained by young adults having more international exposure through social media, thus are more influenced by aesthetics from all over the world. Their cognitive average, regardless of region, likely aligns much more with the Western faces of influencers and celebrities than older adults living in the same region, skewing their preference away from the East Asian composite.^26^

Regarding sexual orientation, the opposite sex preferred the composite most strongly. Attraction to average features of the opposite sex has been thoroughly tested in evolutionary psychology and is at the root of koinophilia. Average features in a mate convey increased chances of passing on one's genes to the next generation and improved viability of one's offspring.^9,27^ Nonbinary respondents rated the composite significantly lower than heterosexual subjects. The same finding was observed in Amaya et al's study of Caucasian composites.^6^ There may be a discrepancy in the LGBTQ+ community's cognitive average when compared with the general population's. Nonbinary people are more likely to live in predominantly LGBTQ+ neighborhoods,^28,29^ proposing that there may be nuance in their beauty preferences due to differing exposure. The specific features that are most attractive to nonbinary individuals are challenging to find in the literature and necessitate further studies into the aesthetics favored by the millions of adults that make up 11% to 26% of the LGBTQ+ adult population in America.^30,31^

There are limitations to our study. The use of only front-facing images does not capture some of the most important features discussed in East Asian Aesthetic surgery. The convexity of the face, prominence of the mandible, and angle of the chin are inaccessible in front-facing images.^32,33^ We were not able to acquire profile shots of the faces from the database; other databases either did not have a sufficient number of standardized photographs of varying views or were not accessible for public research. We appreciate that the addition of other facial angels would give us a more complete assessment of attractiveness. We are currently investigating the use of 3-dimensional averaging technology, as this would dramatically increase our ability to represent the entire face in all dimensions. Examination of different backgrounds was somewhat blunted by the generalization of “Asian” used in our survey. A future study that acknowledges potential biases in country of origin (ie, South Korea, China, Japan, etc) would provide immense insight into more granular differences largely ignored in the West.^21,24,33^ Furthermore, previous studies have disregarded hair and hairline despite their crucial role in facial perception. While acknowledging the potential bias associated with them, we made a deliberate choice to incorporate these aspects into our study due to their proven importance in facial aesthetics.^34,35^ We believe that considering them is essential for a comprehensive assessment of the key facial features contributing to attractiveness.

Our future aims are to expand on the clinical impacts of “cognitive averaging” in the assessment of beauty standards based on personal exposure, by evaluating which features make average faces more attractive and why. We have already initiated a thorough investigation into anthropomorphically mapping and measuring faces of both Caucasian and East Asian descent to elucidate applicable “yardsticks” for surgical decision making. The lack of measurement standards in this manuscript should not undermine the value we have highlighted in discussing patients’ cultural and regional beauty standards in designing preoperative aesthetic goals.

## CONCLUSIONS

This study affirms the utility of using facial composites to guide surgeons in identifying aesthetic standards for patients of East Asian descent. Additionally, we introduced the concept of cognitive averaging and the possible influence social media may have on our aesthetic preferences toward minority ethnicities, suggesting that surgeons may need to consider the cultural setting that the patient lives in, as preferences may vary depending on regional exposure.

## References

[1] Kwak ES . Asian Cosmetic facial surgery. Facial Plast Surg. 2010;26(2):102–109. doi: 10.1055/s-0030-1253497 20446204

[2] Aquino YS , Steinkamp N. Borrowed beauty? Understanding identity in Asian facial cosmetic surgery. Med Heal Care Philos. 2016;19(3):431–441. doi: 10.1007/s11019-016-9699-0 26983846

[3] Wong JK . Aesthetic surgery in Asians. Curr Opin Otolaryngol Head Neck Surg. 2009;17(4):279–286. doi: 10.1097/MOO.0b013e32832cbd04 19602932

[4] McCurdy JA Jr . Considerations in Asian cosmetic surgery. Facial Plast Surg Clin North Am. 2007;15(3):387–397. doi: 10.1016/j.fsc.2007.05.001 17658435

[5] Vegter F , Hage JJ. Clinical anthropometry and canons of the face in historical perspective. Plast Reconstr Surg. 2000;106(5):1090–1096. doi: 10.1097/00006534-200010000-00021 11039382

[6] Amaya J , Wen YE, Shang Z, Jamieson A, Aly A. A crowdsourced evaluation of facial averageness and attractiveness. Aesthet Surg J. 2022;43:(1):NP1–NP11. doi: 10.1093/asj/sjac163 35710301

[7] Langlois JH , Roggman LA. Attractive faces are only average. Psychol Sci. 1990;1(2):115–121. doi: 10.1111/j.1467-9280.1990.tb00079.x

[8] Trujillo LT , Jankowitsch JM, Langlois JH. Beauty is in the ease of the beholding: a neurophysiological test of the averageness theory of facial attractiveness. Cogn Affect Behav Neurosci. 2014;14(3):1061–1076. doi: 10.3758/s13415-013-0230-2 24326966 PMC4053512

[9] Rhodes G . The evolutionary psychology of facial beauty. Annu Rev Psychol. 2006;57(1):199–226. doi: 10.1146/annurev.psych.57.102904.190208 16318594

[10] Schein SS , Trujillo LT, Langlois JH. Attractiveness bias: a cognitive explanation. Behav Brain Sci. 2017;40:e43. doi: 10.1017/S0140525X16000649 28327248

[11] Sharabi SE , Hatef DA, Hollier LH Jr. Facial attractiveness: is the whole more than the sum of its parts? Aesthetic Surg J. 2010;30(2):154–160. doi: 10.1177/1090820X10369370 20442090

[12] Armijo BS , Brown M, Guyuron B. Defining the ideal nasolabial angle. Plast Reconstr Surg. 2012;129(3):759–764. doi: 10.1097/PRS.0b013e3182402e12 22090249

[13] Suhk J , Park J, Nguyen AH. Nasal analysis and anatomy: anthropometric proportional assessment in Asians—aesthetic balance from forehead to chin, part I. Semin Plast Surg. 2015;29(4):219–225. doi: 10.1055/s-0035-1564817 26648801 PMC4656173

[14] Alley TR , Cunningham MR. Article commentary: averaged faces are attractive, but very attractive faces are not average. Psychol Sci. 1991;2(2):123–125. doi: 10.1111/j.1467-9280.1991.tb00113.x

[15] Langlois JH , Roggman LA, Musselman L. What is average and what is not average about attractive faces? Psychol Sci. 1994;5(4):214–220. doi: 10.1111/j.1467-9280.1994.tb00503.x

[16] Halberstadt J , Rhodes G. The attractiveness of nonface averages: implications for an evolutionary explanation of the attractiveness of average faces. Psychol Sci. 2000;11(4):285–289. doi: 10.1111/1467-9280.00257 11273386

[17] Halberstadt J , Rhodes G. It's not just average faces that are attractive: computer-manipulated averageness makes birds, fish, and automobiles attractive. Psychon Bull Rev. 2003;10(1):149–156. doi: 10.3758/bf03196479 12747502

[18] Perrett DI , Lee KJ, Penton-Voak I, et al Effects of sexual dimorphism on facial attractiveness. Nature. 1998;394(6696):884–887. doi: 10.1038/29772 9732869

[19] Nomura M , Motegi E, Hatch JP, et al Esthetic preferences of European American, Hispanic American, Japanese, and African judges for soft-tissue profiles. Am J Orthod Dentofac Orthop. 2009;135(4):S87–S95. doi: 10.1016/j.ajodo.2008.02.019 19362272

[20] Chen T , Lian K, Lorenzana DJ, Shahzad N, Wong R. Occidentalisation of Beauty Standards: Eurocentrism in Asia. Published online. 2020. doi: 10.5281/ZENODO.4325856

[21] Gao Y , Niddam J, Noel W, Hersant B, Meningaud JP. Comparison of aesthetic facial criteria between Caucasian and East Asian female populations: an esthetic surgeon's perspective. Asian J Surg. 2018;41(1):4–11. doi: 10.1016/j.asjsur.2016.07.007 27630035

[22] Jeong S , Lemke BN, Dortzbach RK, Park YG, Kang HK. The Asian upper eyelid: an anatomical study with comparison to the Caucasian eyelid. Arch Ophthalmol. 1999;117(7):907–912. doi: 10.1001/archopht.117.7.907 10408455

[23] Albornoz CR , Bach PB, Pusic AL, et al The influence of sociodemographic factors and hospital characteristics on the method of breast reconstruction, including microsurgery: A U.S. population-based study. Plast Reconstr Surg. 2012;129(5):1071–1079. doi: 10.1097/PRS.0b013e31824a29c5 22544091

[24] Rhee SC , Lee SH. Attractive composite faces of different races. Aesthetic Plast Surg. 2010;34(6):800–801. doi: 10.1007/s00266-010-9606-7 20953953

[25] Naini FB , Cobourne MT, McDonald F, Wertheim D. Submental-cervical angle: perceived attractiveness and threshold values of desire for surgery. J Maxillofac Oral Surg. 2016;15(4):469–477. doi: 10.1007/s12663-015-0872-4 27833339 PMC5083688

[26] Griffey JAF , Little AC. Infant's visual preferences for facial traits associated with adult attractiveness judgements: data from eye-tracking. Infant Behav Dev. 2014;37(3):268–275. doi: 10.1016/j.infbeh.2014.03.001 24793735

[27] Koeslag JH , Koeslag PD. Koinophilia. J Theor Biol. 1994;167(1):55–65. doi: 10.1006/jtbi.1994.1049 8176954

[28] Lee JGL , Boynton MH, Shook-Sa BE, Wimark T. Is where same-sex couples live a valid measure for where single lesbian, gay, and bisexual people live in population health research? Results from a National Probability Phone Survey, 2017, United States. Ann LGBTQ public Popul Heal. 2020;1(2):96–114. doi: 10.1891/lgbtq-2019-0009 PMC7891493 33615310

[29] Rhodes G , Jeffery L, Watson TL, Clifford CW, Nakayama K. Fitting the mind to the world: face adaptation and attractiveness aftereffects. Psychol Sci. 2003;14(6):558–566. doi: 10.1046/j.0956-7976.2003.Psci_1465.x 14629686

[30] Gates GJ . LGBT data collection amid social and demographic shifts of the US LGBT community. Am J Public Health. 2017;107(8):1220–1222. doi: 10.2105/AJPH.2017.303927 28657780 PMC5508189

[31] Wilson B , Meyer I. Nonbinary LGBTQ Adults in the United States. UCLA: the Williams Institute; 2021. Accessed April 14, 2023. https://williamsinstitute.law.ucla.edu/publications/nonbinary-lgbtq-adults-us/

[32] Kuroda S , Sugahara T, Takabatake S, Taketa H, Ando R, Takano-Yamamoto T. Influence of anteroposterior mandibular positions on facial attractiveness in Japanese adults. Am J Orthod Dentofac Orthop. 2009;135(1):73–78. doi: 10.1016/j.ajodo.2006.12.021 19121504

[33] Ioi H , Shimomura T, Nakata S, Nakasima A, Counts AL. Comparison of anteroposterior lip positions of the most-favored facial profiles of Korean and Japanese people. Am J Orthod Dentofac Orthop. 2008;134(4):490–495. doi: 10.1016/J.AJODO.2006.09.070 18929266

[34] Kranz D , Nadarevic L, Erdfelder E. Bald and bad? Exp Psychol. 2019;66(5):331–345. doi: 10.1027/1618-3169/a000457 31603047 PMC7037739

[35] Celikoyar MM , Perez MF, Akbas MI, Topsakal O. Facial surface anthropometric features and measurements with an emphasis on rhinoplasty. Aesthet Surg J. 2022;42(2):133–148. doi: 10.1093/asj/sjab190 33855336

